# The Apple Mobility Trends Data in Human Mobility Patterns during Restrictions and Prediction of COVID-19: A Systematic Review and Meta-Analysis

**DOI:** 10.3390/healthcare10122425

**Published:** 2022-11-30

**Authors:** Artur Strzelecki

**Affiliations:** Department of Informatics, University of Economics in Katowice, 40-287 Katowice, Poland; artur.strzelecki@ue.katowice.pl

**Keywords:** COVID-19, systematic review, mobility data, Apple Mobility Trends Reports

## Abstract

The objective of this systematic review with PRISMA guidelines is to discover how population movement information has epidemiological implications for the spread of COVID-19. In November 2022, the Web of Science and Scopus databases were searched for relevant reports for the review. The inclusion criteria are: (1) the study uses data from Apple Mobility Trends Reports, (2) the context of the study is about COVID-19 mobility patterns, and (3) the report is published in a peer-reviewed venue in the form of an article or conference paper in English. The review included 35 studies in the period of 2020–2022. The main strategy used for data extraction in this review is a matrix proposal to present each study from a perspective of research objective and outcome, study context, country, time span, and conducted research method. We conclude by pointing out that these data are not often used in studies and it is better to study a single country instead of doing multiple-country research. We propose topic classifications for the context of the studies as transmission rate, transport policy, air quality, re-increased activities, economic activities, and financial markets.

## 1. Introduction

Since the emergence of COVID-19, scientists have been working hard to develop models that can predict not only the evolution of the disease, but also the impact of various measures taken [1]. Some of these models want to describe human mobility behavior, which can be unpredictable [2]. The introduction of COVID-19 lockdowns was based on irregular components, mainly due to the different implementations of national legislation restricting the movement of people [3]. In different parts of the world, we observed for the past two years different times of lockdowns, movement restrictions, closure of public places, and many more non-pharmaceutical interventions designed to decrease COVID-19 cases [4].

In recent years, data derived from mobile devices has been increasingly used to study human mobility patterns [5]. This information can be used to understand better how individuals and groups move within cities, as well as to predict future trends in mobility [6]. Such data have the potential to be particularly useful in the context of public health, as it can provide insights into how diseases are spread and allow for more targeted interventions [7]. The use of mobility data for predicting disease patterns is not a new concept; similar approaches have been used previously for epidemics such as influenza [8] and dengue fever [9]. However, the current COVID-19 pandemic has highlighted the need for more real-time data in order to track the spread of the disease and identify areas at risk. Mobile phone data provide a unique opportunity in this regard, as they offer near-complete coverage of urban populations and can be accessed relatively quickly.

The coronavirus pandemic has resulted in a significant increase in the use of mobility data to track the spread of the virus [10]. These data are collected from a variety of sources, including mobile phone apps, GPS devices, and social media platforms [11]. The data are then used to create maps and visualizations that show the patterns of virus spread [12]. These data have been used to track the spread of the virus in real-time and to predict future trends. They have also been used to identify high-risk areas and to inform public health decisions [13]. For example, mobility data were used to identify the early epicenters of the pandemic in China, France, Italy, and the UK [14,15]. These data are also being used to assess the effectiveness of social distancing measures. The use of mobility data has limitations, however. The data are sometimes incomplete and biased [16]. For example, they do not always accurately reflect the movements of people who do not have a smartphone or who do not use apps and social media [17]. Additionally, these data are subject to change and can be challenging to interpret. Despite these limitations, mobility data are a valuable tool for understanding the spread of the coronavirus. These data can be used to save lives by identifying hot spots and informing public health decisions [18].

Several ICT companies, such as Google and Apple, share reports on the mobility of the population, using new technologies that are an integral part of their products or services, daily [19]. They provide movement data from smartphones running mobile applications such as Google Maps or Apple Maps to identify changes in people’s mobility due to COVID-19 [20]. These data cover a large proportion of the population. They can be considered to provide very reliable results, especially if one uses a dataset of both companies that cover most of the world. Several studies have demonstrated a strong relationship between COVID-19 cases and people’s mobility, identifying commuting behavior as a spatial determinant of COVID-19 patterns [21,22,23,24,25,26,27,28]. These studies demonstrate the potential of mobility data for understanding population-level movements and identifying areas of high risk.

There is not much scientific research in mobility data analysis, particularly covering a more extended period and data from a larger region. This systematic review attempts to organize current knowledge on the use of mobility data from cell phones during restrictions and lockdowns caused by COVID-19. In both cases, the Google [29] and Apple [30] companies have already stopped publishing new data on mobility. Data are no longer being updated as of 15 October 2022 in case of Google, and 15 April 2022 in case of Apple. All historical data will remain publicly available. This two-year (2020–2022) period is now closed and data will not change, allowing published studies based on this mobility data to be reviewed. As we continue to grapple with the COVID-19 pandemic, it is likely that mobility data will play an even greater role in prediction and prevention efforts. By understanding past patterns of human mobility, we can begin to anticipate where outbreaks may occur and take steps to prevent them from happening. In a time when reliable information is more critical than ever, mobility data provides a valuable tool for helping us navigate this rapidly changing landscape.

The objective of this review is to discover how population movement information has epidemiological implications for the spread of COVID-19. In many studies using cellphone data, the authors found that population movements significantly explain geographic patterns of COVID-19 transmission [14,31]. During the COVID-19 pandemic, human mobility had changed significantly. In order to understand the impact of the coronavirus disease on human mobility, large-scale and fine-grained mobility data sets are needed.

## 2. Materials and Methods

The review will present research methods used for mobility data exploration, the context of the study, the geographical regions studied, and the outcomes presented in the literature. It will help future studies to be built on this review. The method used in this systematic literature review follows the Preferred Reporting Items for Systematic Reviews and Meta-Analyses (PRISMA 2020) guidelines [32,33]. The eligibility criteria for inclusion in the review is the use of the Apple Mobility Trends Reports. After a preliminary search, we decided to choose one set of mobility data published by a large ICT company: Apple. We select for inclusion studies that used this data. The other inclusion criterion is context. We seek studies that are about COVID-19 mobility patterns during the pandemic time. Another inclusion criterion was for the study to be published in the form of an article or conference paper. We excluded book chapters.

We have searched for relevant studies with the use of the Web of Science database provided by Clarivate and the Scopus database provided by Elsevier as information sources. We did the search in November 2022. The search strategy was using the following search query in Web of Science (WoS): “*ALL = (apple mobility) AND ALL = (covid-19)*”. ALL means that we search in all fields. For the Scopus database, we used the following query: *(TITLE-ABS-KEY (apple AND mobility) AND ALL (covid-19)).* The search strategy resulted in 45 records from WoS and 46 documents from Scopus.

Since the initial number of records for the further selection process started at 91, we decided to check them manually without automation tools. The author screened all works by reading the title and abstract and checking the content of the paper to determine whether it could be included in the review.

The study for inclusion needs to meet three criteria:

1. The study uses data from Apple Mobility Trends Reports.
2. The context of the study is about COVID-19 mobility patterns.
3. Work is published in a peer-reviewed venue in the form of an article or conference paper in English.

The exclusion criteria are:

1. Missing information about the studied country or period.
2. Lack of explained method of how data was used.
3. Lack of information about data use.
4. No COVID-19 context.

The first inclusion criterion is the use of Apple Mobility Trends Reports. Apple’s Mobility Trends Report is based on data sent from users’ devices to the Maps app service. Apple data was published on a daily basis from 13 January 2020 to 14 April 2022 and reports daily changes in requests for directions on the Maps app by three transportation types (driving, transit, and walking) for several spatial levels, such as countries/regions, sub-regions, and cities. Data for 11–12 May 2020, 12 March 2021, and 21 March 2022 is not available and appear as blank columns in the data set. Usually, in the studies, missing values were set as an average of the values for the day before or after. The CSV file shows a relative volume of directions requests per country/region, sub-region, or city compared to a baseline volume on 13 January 2020 [30]. Apple defined day as midnight-to-midnight, Pacific time. Cities are defined as the greater metropolitan area, and their geographic boundaries remain constant across the data set. In many countries/regions, sub-regions, and cities, the relative volume has increased since 13 January, consistent with normal, seasonal usage of Apple Maps. Day-of-week effects are important to normalize as someone uses this data.

Apple provides no information regarding how many users or proportions of Apple users used this function. Data that is sent from users’ devices to the Maps service is associated with random, rotating identifiers, so Apple does not have a profile of individual movements and searches. Apple Maps has no demographic information about our users, so it cannot be made any statements about the representativeness of usage against the overall population. This data was generated by counting the number of requests made to Apple Maps for directions in select countries/regions, sub-regions, and cities. Data that is sent from users’ devices to the Maps service is associated with random, rotating identifiers, so Apple does not have a profile of movements and searches. The availability of data in a particular country/region, sub-region, or city is based on a number of factors, including minimum thresholds for direction requests per day. As of 14 April 2022, Apple is no longer providing COVID-19 mobility trends reports.

The second inclusion criterion is that the context of the study is about COVID-19 mobility patterns. People’s mobility patterns have changed dramatically since the outbreak of COVID-19. It was observed that people were traveling much shorter distances than they were before the pandemic. There has also been a significant decrease in the number of people traveling by car. The number of people using public transport has also decreased. The changes in mobility patterns are likely to have a significant impact on the way cities are designed and operated in the future. The third inclusion criterion is that the report is published in a peer-reviewed journal in the form of an article or presented and published as a conference paper in English. The data collection process was done solely by the author.

The risk of bias was assessed by using the adapted CONSORT scale, which is an 8-item version of the original CONSORT Statement [34]. The CONSORT scale is a quality scale that assesses the quality of randomized controlled trials. The scale has eight domains: title, abstract, introduction, methods, results, discussion, conclusion, and funding. Each domain is rated as 0 (poor quality), 1 (fair quality), 2 (good quality), or 3 (excellent quality). Studies that met fewer than 12 points of the adapted CONSORT criteria were considered to be of low methodologic quality, studies that met 12 to 18 points of the criteria were considered to be of moderate methodologic quality, and studies that met >18 points of the criteria were considered to be of high methodologic quality. Each work was reviewed manually, and collected data was organized in a matrix [35].

## 3. Results

**Figure 1:**
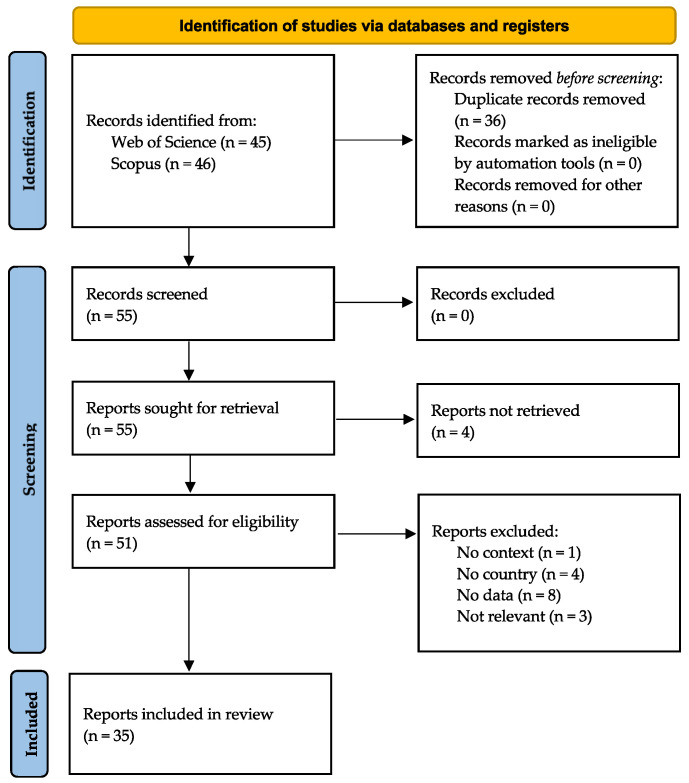
PRISMA flow diagram.

The PRISMA flow diagram in Figure 1 shows the flow of information through the different stages of a systematic review. It shows the number of records identified, included, and excluded and the reason for the exclusion. From the WoS, we have collected 45 records, and from Scopus, 46 records. After removing 36 duplicates, 55 records were screened based on title and abstract.

At this stage, no record was excluded as all abstracts and titles suggested relevancy for the review. Fifty-five reports were sought for retrieval, and 51 reports were retrieved. Four reports were unable to be retrieved due to a lack of full access to the paper. Fifty-one records were assessed for eligibility. At this stage, inclusion and exclusion criteria were applied. Based on the four exclusion criteria, 16 reports were excluded. One report had no COVID-19 context [36], four did not point to the geographical region where it was conducted [37,38,39,40], eight studies did not describe how the mobility data was used [41,42,43,44,45,46,47,48], and three were not relevant [49]. Thirty-five reports were left in the review.

Table 1 presents the overview of included studies. Thirty-five reports were published in the period of 2020 to 2022. In 2020, nine reports were available, in 2021, the number of published papers increased to 22, and in the current year 2022, four papers were included. The most referenced journal is JMIR Public Health and Surveillance, where three reports were published, and Environmental Research Letters, along with the Journal of Medical Internet Research who published two reports each.

There is a significant imbalance in the type of published papers as articles in journals compared to conference proceedings. Thirty-four reports are published in peer-reviewed journals, whereas only one report comes from conference proceedings. There are several reasons why scientific journals were more often chosen than conference proceedings. First, journals are more permanent than conference proceedings. They are more likely to be indexed and searchable, and they are more likely to be read by future generations of scholars. Second, journals are more selective than conference proceedings. They typically have a higher quality bar, which means that only the best and most important research is published in them. Third, journals are more likely to be peer-reviewed than conference proceedings. This means that the research has been vetted by experts in the field, and the journal editors have more confidence in its quality. Finally, journals are more likely to be cited than conference proceedings. This is because they are seen as more authoritative and because they reach a wider audience.

**Figure 2:**
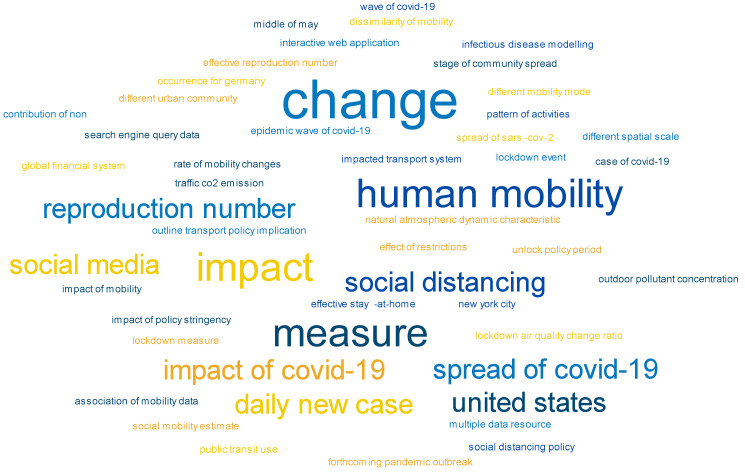
Word cloud of the most important objective and goals based on Table 2.

Table 2 presents the aims of the studies. In the presented research goals, authors are looking at different ways to see how different effective measures are at slowing the spread of COVID-19. This includes looking at how people’s mobility patterns change when there are restrictions in place, how air pollution changes during lockdowns, and how different social distancing policies affect the transmission rate of the virus. In some studies, the purpose was to explore the effects of the lockdown measures on the concentrations of CO_2_ emissions. The studies also looked at the potential of integrating multiple data resource into infectious disease modeling, thereby enhancing the model performance. Reports also examined how CO_2_ emissions responded to the COVID-19 measures at a neighborhood scale. Papers also outlined transport policy implications for developing megacities as a resilience and mitigation strategy to forthcoming pandemic outbreaks and other disruptions. These works are looking at different ways to stop the spread of COVID-19. They are looking at how people are moving around and what effect this has on the virus. They are also looking at how different countries are handling the pandemic and what effect this has on the virus. Figure 2 presents a word cloud to visualize the most important text data in Table 2.

Table 3 presents different dimensions of the studies. We have extracted the following dimensions: country or regions for which the study was conducted, the context of the study, in other words, what is the main topic where the mobility data was used. We also retrieved the timespan for the reports, determining for what period of time the data was analyzed. The last dimension is the scientific method used for conducting the research.

Countries. The reviewed studies we observed studied mobility in a different number of countries. Six studies were multiple country studies, where the number of analyzed countries ranged from 17 to 56. In four of them, there is a list of countries, while two did not contain the exact names of countries, but one has a map of Europe and the USA. In six studies, the number of studied countries ranged from two to five. Twenty-three studies focus on only one country; however, in some one-country reports, presented data was limited to one large megacity. The studied megacities are New York City (USA) in two reports, London (UK), Toronto (Canada), Manila (Philippines), São Paulo (Brazil), Kyoto (Japan) and Delhi (India). The most occurring countries in the reviewed reports are the USA (at least 12 studies), Japan (at least 5 studies), Germany (at least 9 studies), United Kingdom (at least 8 studies), Singapore (at least 3 studies), and the Philippines (2 studies).

Topics: The reported studies were characterized by the topic they point to. The extracted topics are concentrated on transmission rate (13), transport policy (10), air quality (8), re-increased activities (2), economic activities (1), and financial markets (1).

Transmission rate: There is still much uncertainty surrounding the true transmissibility of COVID-19, but estimates suggest that it is more easily spread than originally thought. The transmission rate in the reviewed studies is usually presented as the reproductive number (R) [55,56]. The (R) is considered even higher in some settings, such as crowded living conditions and healthcare settings. This is of particular concern because the virus appears to be highly contagious even before people develop symptoms, which means that it can spread rapidly through a population before anyone knows they are sick. Mobility restrictions can help to reduce the spread of COVID-19. Several studies found that lockdowns led to a significant decrease in the number of new cases. The lockdowns were most effective when combined with other measures, such as temperature checks, mask-wearing, and contact tracing [74]. It is important to note that the lockdowns were lifted after some time, and the number of new cases began to rise again. This highlights the importance of maintaining strict measures for as long as necessary to prevent a resurgence of the disease. The bottom line is that COVID-19 is a highly contagious disease that can spread rapidly through a population. Mobility restrictions can help reduce the spread, but they must be combined with other non-pharmaceutical interventions.

Transport policy: Since the outbreak of the COVID-19 pandemic, there has been a significant change in how people travel. In many countries, lockdown measures have been put in place, which has resulted in a decrease in the use of public transport and an increase in the use of private cars [63]. There are several reasons for this change. Firstly, people are generally more worried about contracting the virus when they are in close proximity to other people. This means that they are less likely to use public transport, which can be quite crowded. Secondly, many people are now working from home, so they do not need to commute to work. This has led to a decrease in the demand for public transport [68]. Private cars are seen as a safer option for many people as they allow people to avoid close contact with others. Additionally, private cars are often seen as being more comfortable than public transport, which can be quite cramped. The change in travel habits is likely to have some consequences. Firstly, it is expected to increase traffic congestion as more people use private cars. Secondly, it could have an impact on public transport revenues as people switch to using private vehicles. Finally, it could lead to more people working from home in the future, as they no longer need to commute to work [81].

Air quality: Since the COVID-19 pandemic began, there has been much discussion about the effect of lockdowns on air quality. Some research suggests that lockdowns have improved air quality in some places, while other studies find that the effect of lockdowns on air quality is mixed [52]. It is well known that air pollution can have negative effects on human health. Air pollution has been linked to a range of health problems, including respiratory infections, heart disease, stroke, and cancer. There is also evidence that air pollution can worsen the symptoms of COVID-19. The effect of lockdowns on air quality is complex and depends on a number of factors, including the type of lockdown, the location, and the types of emissions that are being reduced [60]. In general, lockdowns that result in less traffic and fewer industrial activities tend to improve air quality. However, in some cases, such as in China, lockdowns have also led to an increase in the burning of coal, which can offset any improvements in air quality. It is clear that the effect of lockdowns on air quality is complex and depends on a number of factors. However, given the evidence linking air pollution to a range of health problems, it is important to monitor the air quality in areas where lockdowns are in place [62].

Re-increased activities: Since the outbreak of COVID-19, the world has been in a state of lockdown to prevent the spread of the virus. However, as the number of cases begins to decline, many countries are starting to ease their restrictions and allow people to begin going out and about again. This has led to a re-increase in activities, both in terms of work and leisure. For many people, returning to work has been a welcome relief after months of being stuck at home. It has also been a chance to reconnect with colleagues and customers after a long period of isolation. However, the return to work has also brought with it some challenges, such as how to maintain social distancing in the workplace and how to deal with the increased risk of infection [58]. Similarly, the return to leisure activities has also been mixed. For some, it has been a chance to finally get out and about after being cooped up for so long. However, it has been a source of anxiety for others as they worry about contracting the virus. Overall, the re-increase in activities after the COVID-19 lockdowns has been a mixed experience. For some, it has been a chance to return to normalcy, while for others, it has been a source of anxiety and worry [59].

Economic activities: Decreased mobility and lockdowns have significantly impacted the global economy, with businesses forced to close and people losing their jobs. The lockdowns have particularly severely impacted the tourism and hospitality industries, as people cannot travel [51]. This has led to a decrease in demand for goods and services and a consequent reduction in production. The reduction in production has had a knock-on effect on the rest of the economy, as businesses rely on each other for inputs and outputs. This has led to a decrease in economic activity and a rise in unemployment. The lockdowns have also impacted people’s personal finances, as many have lost their jobs or had their hours reduced. This has led to a decrease in consumer spending, further contributing to the reduction in economic activity.

Financial markets: Lockdowns have had a profound impact on financial markets. Equity markets around the world have seen sharp declines. The economic impact of the pandemic has been severe, with global GDP growth to contract by around 3% in 2020. This has led to a significant increase in risk aversion among investors, with safe-haven assets such as government bonds and gold seeing strong demand. The outlook for financial markets remains uncertain in the short term, but there are reasons to be optimistic in the longer term. The actions of central banks and governments have been supportive of markets, and the global economy is expected to recover once the pandemic is brought under control [79].

Time span: All the reported studies used data starting the year 2020. Fourteen reports used the data from the beginning of data availability, the 13 January 2020. As it was explained, there is a period before the pandemic occurred to set the baseline. Even though some studies were published in 2021 and 2022, this is an effect of the sometimes long peer-review process, or publishing first online and then assigning the paper the issue and volume in the following year.

Methods: The reviewed studies used different statistical methods to assess the results. Among these methods, the most often were descriptive statistics, Pearson’s correlation (coefficient), and regression analysis. Other methods were used to create models for prediction [1,73,74] and Granger causation [38,40].

**Figure 3:**
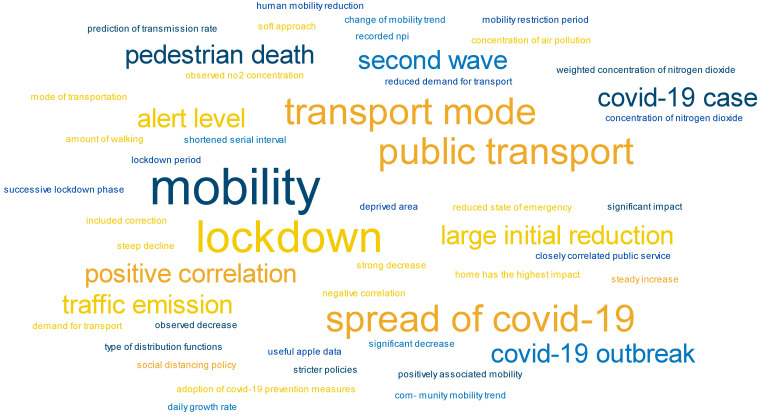
Word cloud of the most important outcomes based on the Table 2.

Table 4 presents the outcomes of the studies. The lockdown has reduced the spread of COVID-19 by decreasing the number of routing requests across all three modes of transportation available in Apple Mobility Trends Data. Additionally, the lockdown has caused a decrease in outdoor air pollution levels. Traffic emissions have decreased significantly since the start of the coronavirus pandemic. All transport modes have seen a decrease in emissions, and public transport has seen the most significant reduction. There is a positive correlation between mobility and the spread of COVID-19, which means that the more people move around, the more the virus will spread. Containment delays and serial intervals were shortened over time, so the virus spread faster than expected. Air pollution concentrations decreased during the mobility restrictions period compared to the same period in 2019. Separately recorded NPIs such as school closure and closure of businesses and public services were closely correlated, both in timing and occurrence. Figure 3 presents a word cloud to visualize the most important text data in Table 4.

## 4. Discussion

This paper reviewed thirty-five studies dedicated to the use of Apple Mobility Trends Reports during the COVID-19 pandemic. The objective of the paper was to compare the results of the studied papers and to discover how population movement information has epidemiological implications for the spread of COVID-19. We searched such research databases as Web of Science and Scopus, finding papers in which Apple data was used. We have applied several inclusion and exclusion criteria.

Firstly, we can state that the use of Apple data is not so common in comparison to the total number of published studies about mobility. COVID-19 has resulted in a drastic change in mobility patterns globally. While some countries have experienced a complete lockdown, others have only implemented partial restrictions [85]. This has resulted in a wide variety of data sources for researchers to study mobility during the pandemic. Except for Apple Mobility Trends Reports, one of the most comprehensive data sources is Google Mobility Reports, which uses aggregated location data from Google Maps to provide insights into changes in travel patterns [86]. There are also a number of government-issued data sources that collect data from state and local transportation agencies [87]. Finally, private companies such as Uber and Lyft have also released data on changes in travel patterns during the pandemic [88]. Overall, the wealth of data available on COVID-19 mobility patterns provide a unique opportunity for researchers to study the impacts of the pandemic on travel behavior.

Secondly, the majority of the reports presented data only for one country. Using mobility data from a single country during the COVID-19 pandemic can provide insights that are not possible when considering data from multiple countries. By looking at a single country’s data, it is possible to track the movement of people within the country and understand how the pandemic has affected different areas. This information can then inform policy decisions about best responding to the pandemic. There are several advantages to using mobility data from a single country. It is easier to compare data from different regions within a country when the data are from a single country [89]. There can be significant variations in mobility patterns between different countries. Using data from a single country allows for a more detailed analysis of the impact of the pandemic on different areas. In contrast, data from multiple countries are often aggregated, making it difficult to see the impact of the pandemic at a more granular level. Data from a single country allow for a more timely analysis of the pandemic, whereas data from multiple countries often take longer to collect and analyze [90]. Data from one country can provide insights into how the pandemic evolves over time. Still, data from numerous countries are often static, making it difficult to see how the pandemic changes over time [91]. It helps to identify potential hotspots for the pandemic because data from multiple countries are often aggregated, making it challenging to identify areas where the pandemic is spreading.

### 4.1. Methodological Limitations in the Reviewed Studies

Identified limitations in the reviewed studies are around the lack of evidence on the effectiveness of mobility restrictions in reducing the spread of COVID-19. The existing evidence is of low quality and is subject to many methodological limitations. The first limitation is the lack of randomization in the studies. This means it is impossible to say with certainty that the observed effects are due to the mobility restrictions and not due to other factors such as changes in people’s behavior. The second limitation is the small number of studies that have been conducted. This means that the results may not be representative of the population as a whole. The third limitation is the short duration of the studies. This means that the long-term effects of mobility restrictions are not known. The fourth limitation is the lack of data on the intensity and duration of the mobility restrictions. This means that it is impossible to say with certainty how effective the restrictions are in reducing the spread of COVID-19. The fifth limitation is the lack of data on compliance with mobility restrictions. This means it is impossible to say with certainty how well the restrictions are being followed.

### 4.2. Limitations of This Systematic Review

The systematic literature review (SLR) is a powerful tool for evidence-based decision-making in public health. However, this SLR has several limitations that should be considered when using it to inform policy decisions on mobility restrictions during the COVID-19 pandemic. First, the SLR only includes studies that have been published in peer-reviewed journals in English. This means that it excludes grey literature, which can often be equally valuable for policy purposes, and that the evidence base is biased towards studies from high-income countries. Second, the SLR only looks at studies that meet pre-specified inclusion criteria. This can lead to bias if the inclusion criteria are not well designed or important studies are excluded. Third, the SLR relies on the availability of published studies, which can introduce biases and limit the generalizability of the findings. Fourth, the SLR does not always consider the quality of the studies included, which can lead to biased results. Finally, the SLR is often time-consuming and resource-intensive, which can limit its usefulness for policy purposes. Finally, the SLR only includes studies examining the effects of mobility restrictions on the spread of COVID-19. This means that the evidence base does not include studies on the effects of mobility restrictions on other outcomes, such as economic growth or public health. Despite these limitations, the SLR remains the gold standard for undertaking comprehensive and rigorous reviews of the literature. When undertaken correctly, it can provide an invaluable overview of the current evidence base and help to inform decision-making in clinical and policy contexts.

### 4.3. Contribution to the Research

The major contribution of this work is the review of thirty-five papers in which Apple Mobility Trends Reports has been applied to analyze its epidemiological assistance in several countries. In the process of SLR, the author has: (1) synthetically described the research conducted in each paper by presenting the main objective and main outcome in each one; (2) created a coding procedure to compare all the reports in terms of studied geographic region (several countries, one country, or a mega city), the context of the study by pointing main topics, retrieved all the time periods for analyzed data, and synthetically described methods used in the reviewed studies; (3) proposed topic classifications for the context of the studies as transmission rate, transport policy, air quality, re-increased activities, economic activities, and financial markets.

### 4.4. Avenues for Future Research

The past three years have seen a dramatic increase in the use of digital tools to support research on human mobility during lockdowns and other restrictions related to the COVID-19 pandemic. This has included the use of different mobility data such as mobile phone data, social media data, and GPS data. While these data sources have proven to be valuable for understanding patterns of human mobility during the pandemic, there are still many unanswered questions about how best to utilize them. In this paper, we reviewed some key findings from studies that used Apple Mobility Trends Reports to study mobility during the pandemic. We identify three avenues for future research that could help to further our understanding of mobility during COVID-19 restrictions: (1) better methods for incorporating uncertainty into analyses of mobility data; (2) improved methods for dealing with non-random sampling biases in mobility data; and (3) greater use of innovative approaches such as machine learning to analyze large-scale mobility datasets, instead of presenting only descriptive statistics.

## 5. Conclusions

The results of this systematic review suggest that data from Apple Mobility Trends Reports can have important implications for understanding the spread of COVID-19. This systematic review with PRISMA guidelines sought to discover how population movement information has epidemiological implications for the spread of COVID-19. The review included 35 studies published in the period of 2020–2022. The main strategy used for data extraction in this review was a matrix proposal to present each study from the perspectives of research objective and outcome, study context, country, time span, and conducted research method. The main finding is that studies that focus on a single country tend to be more informative than those that attempt to cover multiple countries. This is likely due to the fact that data from different countries can be quite different, making it difficult to compare and interpret results. The review also found that the most commonly studied topics in relation to COVID-19 mobility patterns are transmission rate, transport policy, air quality, re-increased activities, economic activities, and financial markets. Overall, the review showed that population movement information does have epidemiological implications for the spread of COVID-19. The use of mobility data can be a valuable tool for understanding the spread of the virus and for making public health decisions.

## Figures and Tables

**Table 1:** The list of reports included in the systematic review.

ID	Study Reference	Type	Journal/Conference
A1	Jacobsen and Jacobsen, 2020 [50]	Journal	World Medical and Health Policy
A2	Camba and Camba, 2020 [51]	Journal	The Journal of Asian Finance, Economics and Business
A3	Li and Tartarini, 2020 [52]	Journal	Aerosol and Air Quality Research
A4	Djilali et al., 2020 [53]	Journal	Biology
A5	Venter at al., 2020 [54]	Journal	Proceedings of the National Academy of Sciences
A6	Delen et al., 2020 [55]	Journal	JMIR Public Health and Surveillance
A7	Younis et al., 2020 [56]	Journal	JMIR Public Health and Surveillance
A8	Walker and Sulyok, 2020 [57]	Journal	Methods of Information in Medicine
A9	Rieger and Wang, 2020 [58]	Journal	Review of Behavioral Economics
A10	Trasberg and Cheshire, 2021 [59]	Journal	Urban Studies
A11	Velders et al., 2021 [60]	Journal	Atmospheric Environment
A12	Jing et al., 2021 [61]	Journal	Journal of Biomedical Informatics
A13	Velasco, 2021 [62]	Journal	Urban Climate
A14	Hasselwander et al., 2021 [63]	Journal	Sustainable Cities and Society
A15	Harkins et al., 2021 [64]	Journal	Environmental Research Letters
A16	Chung and Chan, 2021 [65]	Journal	PLoS ONE
A17	Oda et al., 2021 [66]	Journal	Environmental Research Letters
A18	Kurita et al., 2021 [67]	Journal	JMIR Public Health and Surveillance
A19	Ye et al., 2021 [68]	Journal	Transportation Research Record
A20	Wijayanto and Wulansari, 2021 [69]	Journal	Journal of Physics: Conference Series
A21	Chapin and Roy, 2021 [70]	Journal	Journal of Geovisualization and Spatial Analysis
A22	Huang et al., 2021 [71]	Journal	International Journal of Digital Earth
A23	Cot et al., 2021 [72]	Journal	Scientific Reports
A24	Munawar et al., 2021 [73]	Journal	Sustainability
A25	Husnayain et al., 2021 [74]	Journal	Journal of Medical Internet Research
A26	Al-Jubory and Al-Shamery, 2021 [75]	Conf.	BICITS’21
A27	Kwok et al., 2021 [76]	Journal	Journal of Medical Internet Research
A28	Rudke et al., 2021 [77]	Journal	Environmental Research
A29	Snoeijer et al., 2021 [78]	Journal	npj Digital Medicine
A30	James and Menzies, 2021 [79]	Journal	Chaos: An Interdisciplinary J. of Nonlinear Science
A31	Redelmeier and Zipursky, 2021 [80]	Journal	American Journal of Lifestyle Medicine
A32	Sun et al., 2022 [81]	Journal	Transportation Research Interdisciplinary Perspectives
A33	Wen et al., 2022 [82]	Journal	New Zealand Economic Papers
A34	Padmakumar and Patil, 2022 [83]	Journal	Cities
A35	Fatima et al., 2022 [84]	Journal	MAPAN

**Table 2:** Main objectives and goals set in each of the reviewed reports.

ID	Research Objective/Goal
A1	To assess the effect of stay-at-home orders on mobility patterns during the early stages of community spread of SARS-CoV-2 in the United States.
A2	To investigate the effects of restrictions in economic activity on the spread of COVID-19 in the Philippines.
A3	To quantify the change in outdoor pollutants concentrations during the lockdown period in Singapore, and to evaluate their associations with mobility trends.
A4	To examine how unreported the COVID-19 cases contribute to the dynamic of the spread of this ongoing pandemic.
A5	To study the effect of social distancing policies on ambient air pollutant concentrations.
A6	To study the effect of social distancing policies on the transmission of the coronavirus disease (COVID-19) pandemic.
A7	To study the correlation between social media and public social mobility in relation to social distancing measures.
A8	To examine the relationship between mobility and COVID-19 case occurrence.
A9	To get an overview of patterns of activities and how they change over time.
A10	To assess changes in activity patterns of different urban communities.
A11	To identify and quantify effects of lockdown measures on concentrations.
A12	To explore potential of integrating multiple data resources into infectious disease modeling to enhance model performance.
A13	To examine how CO_2_ emissions responded to COVID-19 measures at the neighborhood scale.
A14	To outline transport policy implications for developing megacities as a resilience and mitigation strategy to forthcoming pandemic outbreaks and other disruptions.
A15	To quantify changes in U.S. gasoline and diesel consumption throughout the COVID-19 pandemic.
A16	To evaluate impacts of policy stringency and residents’ compliance on time-varying reproduction number.
A17	To show the impact of COVID-19 on traffic CO_2_ emissions over the first six months of 2020 in Japan.
A18	To investigate the associations of mobility data provided by Apple Inc and to estimate an effective reproduction number.
A19	To determine the effect of social media on human mobility before and after the COVID-19 outbreak, and its impact on personal vehicle and public transit use in New York City.
A20	To quantify the correlation between human mobility and the daily new cases of COVID-19.
A21	To create an interactive web application to visualize in near-real time the relationship between the COVID-19 pandemic and human mobility, as well as the impact of governmental policies.
A22	To examine the similarity and dissimilarity of mobility from various sources, and the luxury nature of social distancing in the USA during the COVID-19 pandemic, highlighting the disparities in mobility dynamics from lower-income and upper-income groups.
A23	To identify, quantify, and classify different degrees of social distancing and their effect on the first wave of the COVID 19 pandemic in Europe and the United States.
A24	How the transport system is impacted because of the policies adopted by the Australian government for the containment of COVID-19.
A25	To analyze whether search engine query data are important variables for predicting new daily COVID-19 cases and deaths in short- and long-term periods.
A26	To analyze whether search engine query data are important variables that should be included in the models predicting new daily COVID-19 cases and deaths.
A27	To examine the impact of mobility on the spread of COVID-19.
A28	To characterize the epidemiology of the first two epidemic waves of COVID-19 in Hong Kong.
A29	To offer an analysis that puts the period under the influence of the pandemic restrictions in a broader context and that considers the natural atmospheric dynamics characteristics.
A30	To investigate the proportional contribution of Non-Pharmaceutical Interventions to the magnitude and rate of mobility changes.
A31	To determine if the reduction in pedestrian deaths was proportional to the reduction in mobility.
A32	To quantify the impacts of multiple non-pharmaceutical interventions on activity trends across the timeline of the ongoing COVID-19 pandemic in Japan.
A33	To quantify the impact of COVID-19 on changes in community mobility and variation in transport modes.
A34	To analyze changes in usage of different mobility modes during the national lockdown and unlock policy periods across six Indian cities.
A35	To explore the pre-lockdown and during lockdown air quality change ratio along with meteorological effects.

**Table 3:** Studied countries, context, timespan, and methods used for the reviewed studies.

ID	Studied Country (es)	Context	Date Start	Date Stops	Used Method
A1	USA	Transport policy	13 January 2020	29 March 2020	Descriptive statistics
A2	Philippines	Economic activities	17 February 2020	11 September 2020	Least squares regression
A3	Singapore	Air quality	20 March 2020	11 May 2020	Spearman’s rank correlation
A4	Algeria, Egypt, and Morocco	Transmission rate	18 March 2020	10 June 2020	Mathematical model
A5	34 countries	Air quality	13 January 2020	15 May 2020	Linear regression
A6	26 countries (ECDC)	Transmission rate	28 February	17 April 2020	Machine learning regression algorithm
A7	USA	Transmission rate	5 March 2020	5 April 2020	Pearson correlations
A8	Germany	Transmission rate	27 January 2020	18 August 2020	Generalized additive model
A9	France, Germany, UK and the USA	Re-increased activities	13 January 2020	22 April 2020	Pearson correlations
A10	UK, London	Re-increased activities	9 March 2020	13 July 2020	Regression analysis
A11	The Netherlands	Air quality	16 March 2020	10 May 2020	Machine learning (Random forest)
A12	UK, Italy, Spain, France, and Germany	Transmission rate	23 March 2020	30 June 2020	Dynamic model
A13	Singapore	Air quality	13 January 2020	1 June 2020	Flux model
A14	Philippines, Manila	Transport policy	13 January 2020	30 September 2020	Descriptive statistics
A15	USA	Air quality	13 January 2020	31 December 2020	Descriptive statistics
A16	17 countries	Transmission rate	13 January 2020	8 April 2020	Descriptive statistics
A17	Japan	Air quality	13 January 2020	30 June 2020	Emission model
A18	Japan	Transmission rate	10 February	30 June 2020	Polynomial function
A19	USA, NYC	Transport policy	13 January 2020	28 September 2020	Basic difference comparison
A20	Indonesia	Transmission rate	1 March 2020	31 July 2020	Cross-correlation analysis
A21	USA, Japan, and India	Transmission rate	13 January 2020	13 October 2020	Descriptive statistics
A22	Singapore	Transport policy	1 March 2020	29 June 2020	Pearson correlations
A23	22 countries	Transmission rate	1 March 2020	31 May 2020	Correlation
A24	Australia	Transport policy	13 January 2020	31 October 2020	Regression analysis
A25	South Korea	Transmission rate	20 January 2020	31 July 2021	Generalized linear models
A26	Australia, Germany, UK, and USA	Transmission rate	18 February	21 April 2020	Pearson correlations
A27	Hong Kong	Transmission rate	23 January 2020	2 August 2020	Multivariable regression model
A28	Brazil, São Paulo	Air quality	16 March 2020	30 June 2020	Mann–Whitney U test
A29	56 countries	Transport policy	13 January 2020	14 June 2020	Descriptive statistics
A30	20 countries	Financial markets	13 January 2020	30 December 2020	Hierarchical clustering
A31	USA, NYC and Canada, Toronto	Transport policy	13 January 2020	31 December 2020	Descriptive statistics
A32	Japan, Kyoto	Transport policy	15 February 2020	2 April 2021	Regression with ARIMA
A33	New Zealand	Transport policy	15 February 2020	9 July 2020	ARCH model
A34	India	Transport policy	25 March 2020	30 September 2020	Descriptive statistics
A35	India, Delhi	Air quality	15 February 2020	20 June 2020	Descriptive statistics

**Table 4:** List of the main reported outcomes reported in the reviewed studies.

ID	Reported Outcome
A1	The decrease in the use of all three modes of transportation was substantial in all 15 cities, by at least 55 percent, even in states without stay-at-home orders.
A2	The highest impact in reducing the spread of COVID-19 is staying-at-home, followed by visiting transit stations less, less use of public transport, less walking, and less workplace visits.
A3	The lockdown significantly decreased outdoor air pollution when compared with the same period in the previous four years, even with corrections for long time trends in the analysis.
A4	A model to estimate the second wave of the COVID-19 Algeria and Morocco and to project the end of the second wave.
A5	The lockdown events reduced the population-weighted concentration of nitrogen dioxide and particulate matter levels by about 60% and 31% in 34 countries.
A6	Social distancing policies explain approximately 47% of the variation in the disease transmission rates.
A7	Social media tools can be used to assess the effectiveness of social distancing measures.
A8	A negative correlation exists between mobility and confirmed case numbers.
A9	Most areas see a small but steady increase in activity after a steep decline due to the COVID-19 outbreak and lockdown measures.
A10	Activities in deprived areas dominated by minority groups declined less compared to the Greater London average.
A11	The lockdown reduced observed NO_2_ concentrations by 30%, 26%, and 18% for traffic, urban, and rural background locations, respectively.
A12	There was a positive correlation between the average daily change of mobility trend and control rate.
A13	Traffic emissions dropped 41%, but emissions from cooking and metabolic breathing increased 21% and 20%, respectively.
A14	All transport modes experienced significant decreases, with public transport experiencing the largest drop (−74.5 %, on average).
A15	Mobility datasets tend to overestimate traffic reductions in April 2020 (i.e., lockdown period).
A16	The reproduction numbers of COVID-19 surged rapidly at the initial epidemic stage, but declined gradually depending on policy stringency. Human mobility reduction was greater in countries with stricter policies.
A17	During Japan’s state of emergency, traffic emissions were reduced by 23.8% compared to the emission level of the previous year, despite Japan’s soft approach in response to COVID-19.
A18	Apple data are useful for short-term prediction of transmission rate.
A19	In general, mobility trend correlations are negative for both driving and transit categories, especially at the beginning of the COVID-19 outbreak in NYC.
A20	The COVID-19 case daily growth rate is correlated with the human mobility patterns of driving and walking activities on both weekends and weekdays time in Jakarta (province-level) and Indonesia (country-level).
A21	The data suggest a high degree of spatial autocorrelation in mobility and COVID-19 case patterns, meaning that locations near each other share similar patterns.
A22	Counties with higher income tend to more aggressively reduce mobility in response to the pandemic.
A23	A strong decrease in the infection rate is observed two to five weeks after the reduction in mobility.
A24	Study showed reduced demand for transport with the adoption of COVID-19 prevention measures.
A25	GLMs with different types of distribution functions may have been beneficial in predicting new daily COVID-19 cases and deaths in the early stages of the outbreak.
A26	The spread of COVID-19 is positively associated with mobility.
A27	Containment delays and serial intervals were shortened over time.
A28	The Metropolitan Area of São Paulo reached an average decrease of 29% in CO, 28% in NOx, 40% in NO, 19% in SO_2_, 15% in PM2.5, and 8% in PM10 concentrations during the mobility restrictions period compared to the same period in 2019.
A29	Separately recorded NPIs such as school closure and closure of businesses and public services were closely correlated with each other, both in timing and occurrence.
A30	Mobility data and national financial indices exhibited the most similarity in their trajectories, with financial indices responding quicker to surges in COVID-19 cases.
A31	A large initial reduction in pedestrian deaths was found during the lockdown in both New York and Toronto. However, the reduction was not sustained in either city. In New York, the reduction was transient and not statistically significant during the summer and autumn, despite sustained reductions in pedestrian activity.
A32	Policies that restrict mobility can have different effects when an intervention or event occurs multiple times.
A33	The significant impact of Alert Level 4 lockdown on mobility and transport mode variation led to a progressive return to pre-lockdown patterns, with the exception of public transport.
A34	Association investigations through generalized linear mixed-effects models identify income, vehicle registrations, and employment rates at the city level to significantly impact the community mobility trends.
A35	The gradual decrease/increase in concentrations of air pollution was found well correlated with people’s mobility during successive lockdown phases.

## Data Availability

Not applicable.
